# Resveratrol activates MAPK/ERK pathway to regulate oestrogen metabolism in type I endometrial cancer

**DOI:** 10.1186/s12906-024-04509-y

**Published:** 2024-06-11

**Authors:** Qing Wang, Jia-yun Zhou, Li Liu, Ze-yuan Yin, Yan-yu Li, Meng Wang, Jing-bo Zhang, Hui Lu, Xue-yan Zhou, Bei Zhang

**Affiliations:** 1 Department of Obstetrics and Gynecology, Xuzhou Central Hospital, The Xuzhou Clinical College of Xuzhou Medical University, Xuzhou, Jiangsu China; 2 Department of Obstetrics and Gynecology, Graduate School of Bengbu Medical University, Bengbu, China; 3 Jiangsu Key Laboratory of New Drug Research and Clinical Pharmacy, Xuzhou Medical University, Xuzhou, Jiangsu China; 4 Department of Cardiology, The Affiliated Hospital of Xuzhou Medical University, Xuzhou, China; 5 Department of Physical Examination Center, Xuzhou Central Hospital, Xuzhou, China

**Keywords:** Resveratrol, LCMS/MS, Oestrogen homeostasis, MAPK pathway, Network pharmacology, Endometrial cancer

## Abstract

**Objective:**

Endometrial cancer (EC) is an oestrogen-dependent tumour, the occurrence of which is closely related to an imbalance of oestrogen homeostasis. Our previous studies explored the effects of Resveratrol(Res) on oestrogen metabolism. However, systematic research on the exact mechanism of action of Res is still lacking. Based on network pharmacology, molecular docking and animal experiments, the effects and molecular mechanisms of Res on endometrial cancer were investigated.

**Methods:**

The target of Res was obtained from the high-throughput experiment and reference-guided database of TCM (HERB) and the Encyclopedia of Traditional Chinese Medicine (ETCM) databases, and the target of endometrial cancer was obtained by using the Genecards database. Venny map was used to obtain the intersection target of Res in the treatment of endometrial cancer, and the protein interaction network of the intersection target was constructed by importing the data into the STRING database. Then, the drug–disease–target interaction network was constructed based on Cytoscape 3.9.1 software. Gene Ontology (GO) and Kyoto Encyclopedia of Genes and Genomes (KEGG) pathway enrichment analyses were performed for intersection targets using the OmicShare cloud platform. Res and core targets were analysed by molecular docking. EC model mice induced by MNNG were randomly divided into the control group, Res group, MNNG group, MNNG + Res group, and MNNG + Res + MAPK/ERKi group. The protein levels of ERK and p-ERK in the mouse uterus were detected by Western blot. The levels of E1, E2, E3, 16-epiE3, 17-epiE3, 2-MeOE1, 4-MeOE1, 2-MeOE2, 4-MeOE2, 3-MeOE1, 2-OHE1, 4-OHE1, 2-OHE2, 4-OHE2, and 16α-OHE1 in the serum and endometrial tissue of mice were measured by LC‒MS/MS.

**Results:**

A total of 174 intersection targets of Res anti-endometrial cancer were obtained. The signalling pathways analysed by KEGG enrichment included the AGE-RAGE signalling pathway in diabetic complications, the PI3K-Akt signalling pathway and the MAPK signalling pathway. The top 10 core targets were MAPK3, JUN, TP53, CASP3, TNF, IL1B, AKT1, FOS, VEGFA and INS. Molecular docking showed that in addition to TNF, other targets had good affinity for Res, and the binding activity with MAPK3 was stable. Western blot results showed that Res increased the phosphorylation level of ERK and that MAPK/ERKi decreased ERK activation. In the LC-MS/MS analysis, the levels of 2-MeOE1, 2-MeOE2 and 4-MeOE1 in serum and uterine tissue showed a significantly decreasing trend in the MNNG group, while that of 4-OHE2 was increased (*P* < 0.05). The concentrations of 4-MeOE1 in serum and 2-MeOE1 and 2-MeOE2 in the endometrial tissue of mice were significantly increased after Res treatment, and those of 4-OHE2 in the serum and uterus of mice were significantly decreased (*P* < 0.05). Meanwhile, in the MAPK/ERKi intervention group, the effect of Res on the reversal of oestrogen homeostasis imbalance was obviously weakened.

**Conclusion:**

Res has multiple targets and multiple approaches in the treatment of endometrial cancer. In this study, it was found that Res regulates oestrogen metabolism by activating the MAPK/ERK pathway. This finding provides a new perspective for subsequent research on the treatment of endometrial cancer.

**Supplementary Information:**

The online version contains supplementary material available at 10.1186/s12906-024-04509-y.

## Introduction

Endometrial cancer (EC) is the most common gynaecological malignancy in developed countries, the incidence of which is on the rise, and the age of affected patients is decreasing, making this a serious threat to women’s health [1, 2]. Although usually detected in early at stages with more favourable prognoses, individuals with advanced-stage disease have a worse 5-year survival rate, ranging from 47 to 58% (stage III) and from 15 to 17% (stage IV). These data indicate the urgent need to elucidate the molecular alterations associated with endometrial cancer in an effort to identify targets for future prevention and treatment strategies [3]. Type I EC is the most common type of EC. Abnormal accumulation of endogenous oestrogen and its metabolites is a key risk factor for the development of type I EC, and its occurrence is closely related to the imbalance of oestrogen homeostasis [4]. The oestrogen metabolite hydroxy-oestrogen can form adducts with DNA, cause gene mutations, and produce direct genotoxicity [5]. The endogenous conversion of oestrogen to genotoxic metabolites has been reported as an alternative potentially ER-independent mechanism for oestrogen-dependent malignant tumorigenesis [6]. However, the body of literature surrounding this topic is quite sparse because the low levels of oestrogen metabolites makes their measurement difficult. Metabolomics is a powerful high-throughput approach used to identify metabolites or metabolic signatures that are associated with disease development and could help identify novel biological mechanisms involved in pathogenesis [3, 7]. In our previous research, based on the technology platform of liquid chromatography and tandem mass spectrometry (LC–MS/MS), we established a stable and reliable quantitative metabolomic detection method for the oestrogen active substances group [8]. A total of 100 EC patients and 100 healthy subjects were recruited for analysis of the oestrogen active substances group (E1, E2, E3, 16α-OHE1, 2-MeOE1, 3-MeOE1, 4-MeOE1, 2-MeOE2, 4-MeOE2, 2-OHE1, 4-OHE1, 2-OHE2, 4-OHE2, 16-epiE3,17-epiE3), our results showed that the oestrogen homeostasis profile was disrupted in EC patients, with carcinogenic hydroxy-oestrogen (4-OHE1, 2-OHE1, 2-OHE2) significantly accumulated in the serum of these patients. Using OPLS-DA, we noted that 4-OHE1, 2-OHE1, and 2-OHE2 are EC-related disease markers, which further confirmed that hydroxy-oestrogen plays an essential role in the occurrence and development of EC [8]. Therefore, exploring the effective regulation of the oestrogen metabolic pathway and restoring the homeostasis of oestrogen may be an important means to prevent the occurrence of oestrogen-dependent EC.

Res is a phytoestrogen [9] that has excellent antitumour activity, has low cytotoxicity in normal cells, and is easy to obtain from the diet, and there has been research on the anticarcinogenic effects of phytoestrogens [10]. There are 4 main classes of phytoestrogens: isoflavones, stilbenes, coumestans, and lignans. Res belongs to the group of stilbenes and is one of the most actively studied phytoestrogens in this field [11]. Although both cis- and trans-structures have been found in nature, only E-Res exhibits bioactivity. It is widely distributed in grape berry skins and seeds and peanuts, particularly in the dried roots of the plant Polygonum cuspidatum [12–14]. The molecular structure of Res is similar to that of oestrogen 17β-oestradiol and synthetic oestrogen E‐diethylstilbesterol. This structure includes phenolic rings that are essential for binding to ERα and ERβ, as well as hydroxyl groups at specific positions, and this structure has the ability to modulate the biological response of oestrogen by binding to ER [12]. According to spatial structure considerations, Res is expected to interfere with the functioning of 17β‐oestradiol, and hence, Res is involved in reducing the incidence of oestrogen-dependent tumours [15]. In Chinese hamster ovary cells (CHO-K1), it was found that Res binds the two ER receptors (ER alpha and beta) with a similar affinity but with an affinity approximately 7,000 times lower than that of oestradiol [16]. Henry et al. [17] examined the effect of Res administered to female rats in vivo. Although Res did not show high affinity for ERs, it was still able to affect the hypothalamic–pituitary–gonadal axis regulatory genes, affecting the oestrous cycles and inducing gonad hypertrophy in intact animals. In recent studies conducted with both endometrial and mammary cancer cells, scholars have demonstrated that Res has antiproliferative and cancer-protective effects through an ER-independent pathway [18–20]. There has been some experimental and clinical evidence to indicate that Res interferes in the metabolic disposition of oestrogens [18, 21, 22]. Disruption of oestrogen metabolism due to a homeostatic imbalance between activating and protecting enzymes increases the formation of catechol metabolites and their corresponding oestrogenic quinones, which predisposes patients to the development of cancer. Res was shown in several studies to inhibit the formation of catechol derivatives [18, 21, 22]. Our previous research also indicated that Res may be beneficial for restoring oestrogen homeostasis in a breast cancer model and thus slowing the pathological development of breast cancer [23]. Moreover, as demonstrated by our other study conducted with ICR endometrial cancer model mice, due to the imbalance of oestrogen homeostasis in EC mice, Res decreased the levels of the oestrogen genotoxicity metabolite 4-OHE2, suppressed endometrial proliferation, and inhibited the occurrence of EC [24]. These findings provide new evidence to better understand the antitumour activity of Res, but the precise mechanism by which Res restores oestrogen homeostasis remains unclear.

**Fig. 1:**
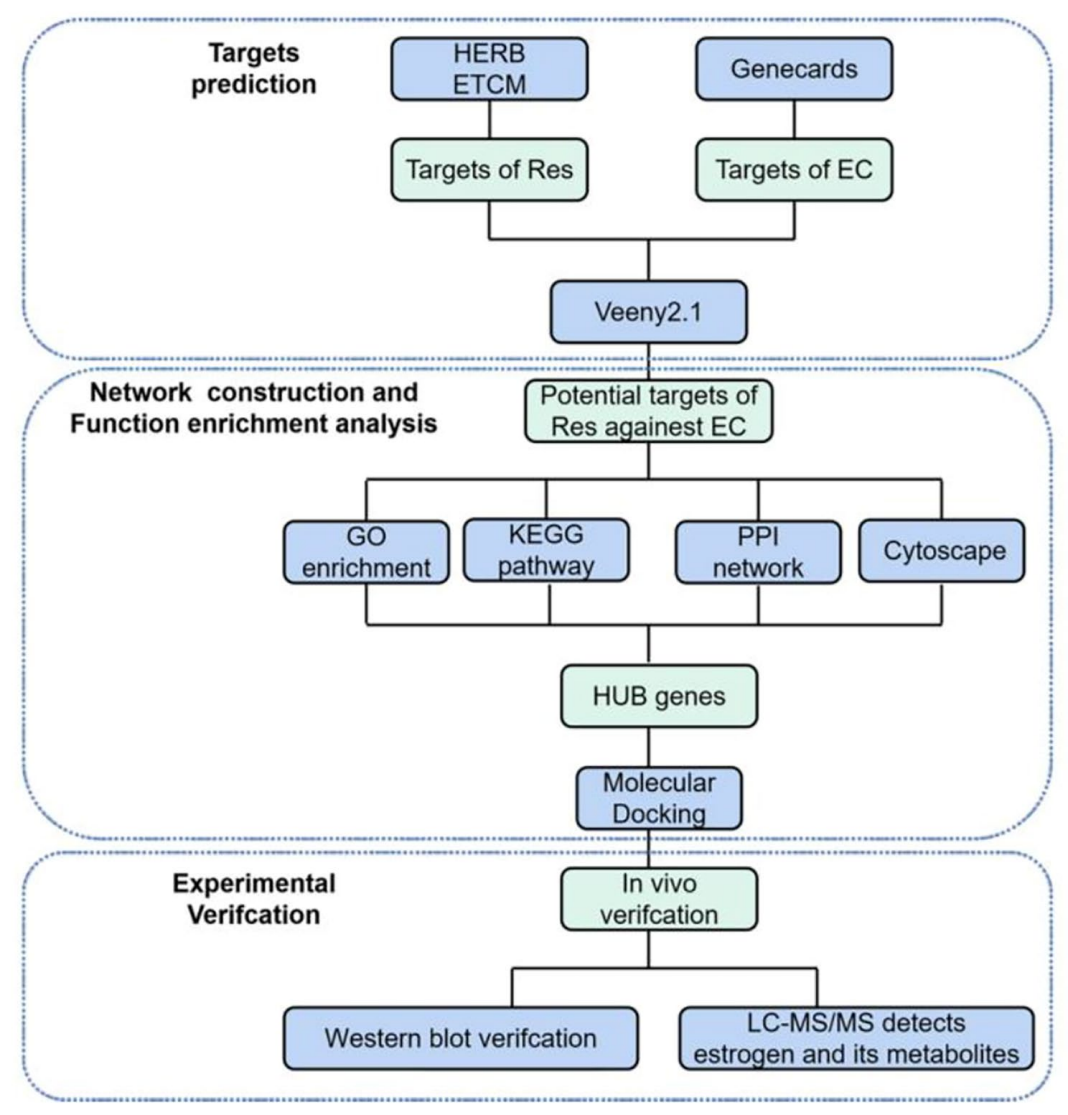
Flowchart of the whole study design

The purpose of our present study was to screen potential targets of Res in regulating oestrogen metabolism using network pharmacological analysis. We also performed a molecular docking study to verify the prediction. Then, we verified the results in EC model mice. In addition, we observed changes in oestrogen and its metabolites in the serum and endometrial tissue of mice under Res and target inhibitor intervention. The whole research process is shown in Fig. 1.

## Materials and methods

### Materials

MNNG (no. R-081 N) and Res (no. M02442) was purchased from J&K Scientific, China. Oestradiol (no. E8875), estrone (no. 1,238,002) and estriol (no. E-074) were purchased from Sigma–Aldrich, USA. 2-Methoxy-oestradiol (no. E2490-000), 4-hydroxy-oestradiol (no. E250-000), 2-hydroxy-oestradiol (no. E2470-000), 16alpha-hydroxy-oestradiol (no. E1250-000), 16-epi-estriol (no. E2570-000), and 17-epi-estriol (no. E2850-000) were purchased from Steraloids, Canada. 4-Methoxyestradiol (no. M262630), 2-hydroxyestrone (no. H941945), 4-hydroxyestrone (no. H941950), 2-methoxyestrone (no. M262520), and 4-methoxyestrone (no. M226135) were purchased from J&K Scientific, China. D5-oestradiol (no. D-5552) was purchased from C/D/N Isotope Corporation, USA. Ulixertinib (HY-15,816) was purchased from MCE, USA.

### Network pharmacology approach

Polygonum cuspidatum was searched for in HERB (http://herb.com/) and ETCM (http://etcm.com/), and related chemical constituents were collected. Then, the OB and DL values were used to screen the effective chemical components, and a total of 13 active components were collected. The collected active ingredients were used to find corresponding targets in the HERB and ETCM systems, with a total of 79 targets. The resulting targets were converted to the corresponding genotypes in the UniProt database (http://www.uniprot.org/). Endometrial cancer genes were searched in Genecards (https://www.genecards.org) databases, and the total genes were obtained after deleting duplicate genes. The Venny 2.1 website was used to draw a Venn diagram and obtain 174 intersection targets. Cytoscape 3.9.1 software was used to construct a “compound-target-disease” network, and a topological network was used to screen out core compounds and important targets. Moreover, GO function and KEGG pathway enrichment analyses were performed. Using OmicShare (https://www.omicshare.com/) to perform biological analysis on the intersection targets, significant biological functions and signalling pathways were obtained. The intersection targets obtained above were entered into the STRING database (https://string-db.org/), and PPI analysis was performed to analyse protein‒protein interactions.

### Molecular docking

To verify the interaction between the core active ingredient Res and the core targets, molecular docking verification was carried out. The structural formula of the active ingredient was downloaded from the PubChem database (https://pubchem.ncbi.nlm.nih.gov/). Chem3D software was used to make the corresponding 3D structure, which was output in mol^*^2 format and then downloaded from the PDB database (http://www.rcsb.org/) to download the pdb format of the core protein domain. PyMOL software was used to dehydrate and dephosphorylate the protein, and AutoDockTools 1.5.6 software was used to convert the active ingredients of the drug and the core protein-encoding gene file pdb to convert the format to pdbqt format and search for the active pocket. Finally, the Vina script was used to calculate the molecular binding energy and display the molecular docking results. At the same time, Discovery Studio 2019 was run to find the docking site, calculate the flexible binding LibDockScore, and import the output molecular docking results into PyMOL software. The molecular docking conformation display was performed. Stable docking was indicated when the binding energy of Vina was ≤ − 5.0 kcall·mol^− 1^, the LibDockScore could find the docking site, and the LibDockScore was > 0. The receptor‒ligand complexes were displayed in 3D and 2D formats for molecular docking results to evaluate the reliability of the bioinformatic analysis predictions.

### Animals and treatment

All animal experimental protocols were approved by the Committee of Xuzhou Medical University on the Ethics of Animal Experiments (202209S029). SPF grade ICR mice, with a body weight of 16 ∼ 20 g, were purchased from Jiangsu Charles River Laboratory Animal Co., Ltd, and raised in the Key Laboratory of New Drugs and Clinical Pharmacy, Xuzhou Medical University Clean animal laboratory, licence number: SCXK (Zhe) 2019-000. During the experiment, mice in each group were given free access to drinking water. All mice were raised in the experimental animal centre (environment: relative humidity 65%, room temperature 25 ± 2℃, relative humidity 55 ± 5%, alternating 12-h light and dark cycles).

A total of 30 mice were randomly divided into five groups with six mice in each group: normal control group (control group), Res group (Res treatment group), model group (MNNG group), model + Res administration group (MNNG + Res group), and model + Res + MAPK/ERK inhibition administration group (MNNG + Res + MAPK/ERK i group). Among them, the MNNG group, MNNG + Res group and MAPK/ERKi group were given MNNG (dissolved in 50% polyethylene glycol for a single dose of 60 mg·kg^− 1^) once a week for 3 weeks. The control group and the Res group were given 50% polyethylene glycol in the uterine cavity for sham operation (the administration volume and times were the same as those of the model group). After the first modelling, the control group and MNNG group were given blank purified diet. The Res group, MNNG + Res group and MNNG + Res + MAPK/ERKi group were given Res-containing (800 mg·kg^− 1^) purified dietary formula feed for 36 weeks. ERKi (ulixertinib) was administered orally at 50 mg·kg^− 1^ BID for 6 weeks. When treatment was finished, the mice were sacrificed by cervical dislocation, and serum and uterine tissue were collected for testing.

### Quantification of oestrogens using liquid chromatography–tandem

#### Mass spectrometry

An appropriate amount of mouse uterine tissue was accurately weighed, placed in 100 g L^− 1^ PBS buffer, and fully homogenized in an ice-water bath to prepare the tissue homogenate at 100 g L^− 1^. In total, 300 μL of homogenate or mouse serum was removed, 10 μL of d5-E2 solution was added (50 nmol·L^− 1^) as the internal standard, the samples was vortex for 1 min, and ethyl acetate was added at a volume to volume (V: V) ratio of 1:3. The sample was vortexed for 10 min and centrifuged at 13,000 r·min^− 1^ for 5 min at 4℃. The upper organic phase was collected and spun dry in a vacuum freeze dryer at 40℃. Then, 50 μL of 0.1 mol·L^− 1^ Na_2_CO_3_/NaHCO_3_ buffer solution (pH 9.0) and 50 μL of 1 g L^− 1^ dansyl chloride acetone solution were added, and the sample was vortexed thoroughly for 1 min. The reaction was heated in a 65 °C oven for 7 min. After cooling, it was centrifuged at 4 °C and 13,000 r·min^− 1^ for 15 min, and the supernatant was transferred into a sample vial. The contents of 11 target analytes in the samples were determined by LC‒MS/MS, and the detection method was determined by the liquid chromatography‒mass spectrometry method established and verified by our research group in the early stage [8]. The standard working curve method was used for internal standards.

### Western blot analyses

Uterine tissue was sufficiently homogenized in RIPA lysis buffer, and total protein was extracted using a commercial extraction kit (P0013B, Beyotime, Shanghai, China). The protein concentration was determined by the BCA protein assay kit (P0013B, Beyotime, Shanghai, China). Subsequently, the prepared protein sample was separated by polyacrylamide–SDS gel electrophoresis and then transferred onto PVDF membranes. After blocking with 5% non-fat dry milk for 1 h, the PVDF membranes were incubated with primary antibodies overnight at 4 °C. The next day, the blots were subsequently incubated with HRP-conjugated secondary antibodies. All experiments were repeated three times. The intensities of the bands were quantified using the Odyssey® CLx (LICOR, USA). ERK1/2 Antibody (AF0155, Affinity Biosciences, USA), Phospho-ERK1/2 (Thr202/Tyr204) Antibody (AF1015, Affinity Biosciences, USA), GAPDH (AP0063, Bioworld, USA), and IRDye 800CW goat anti-rabbit Ig(H + L) (v926-32211, Vicmed, Jiangsu, China) were used in this study.

### Statistical analysis

The collected LC-MS/MS data were quantitatively analysed and processed using MassHunter Quantitative software. Statistical analysis was performed using SPSS 26.0 software. The data are expressed as the means ± SEMs, and comparisons among groups were performed using one-way ANOVA.

## Result

### Common targets of res and type I endometrial cancer screened with network pharmacology

**Fig. 2:**
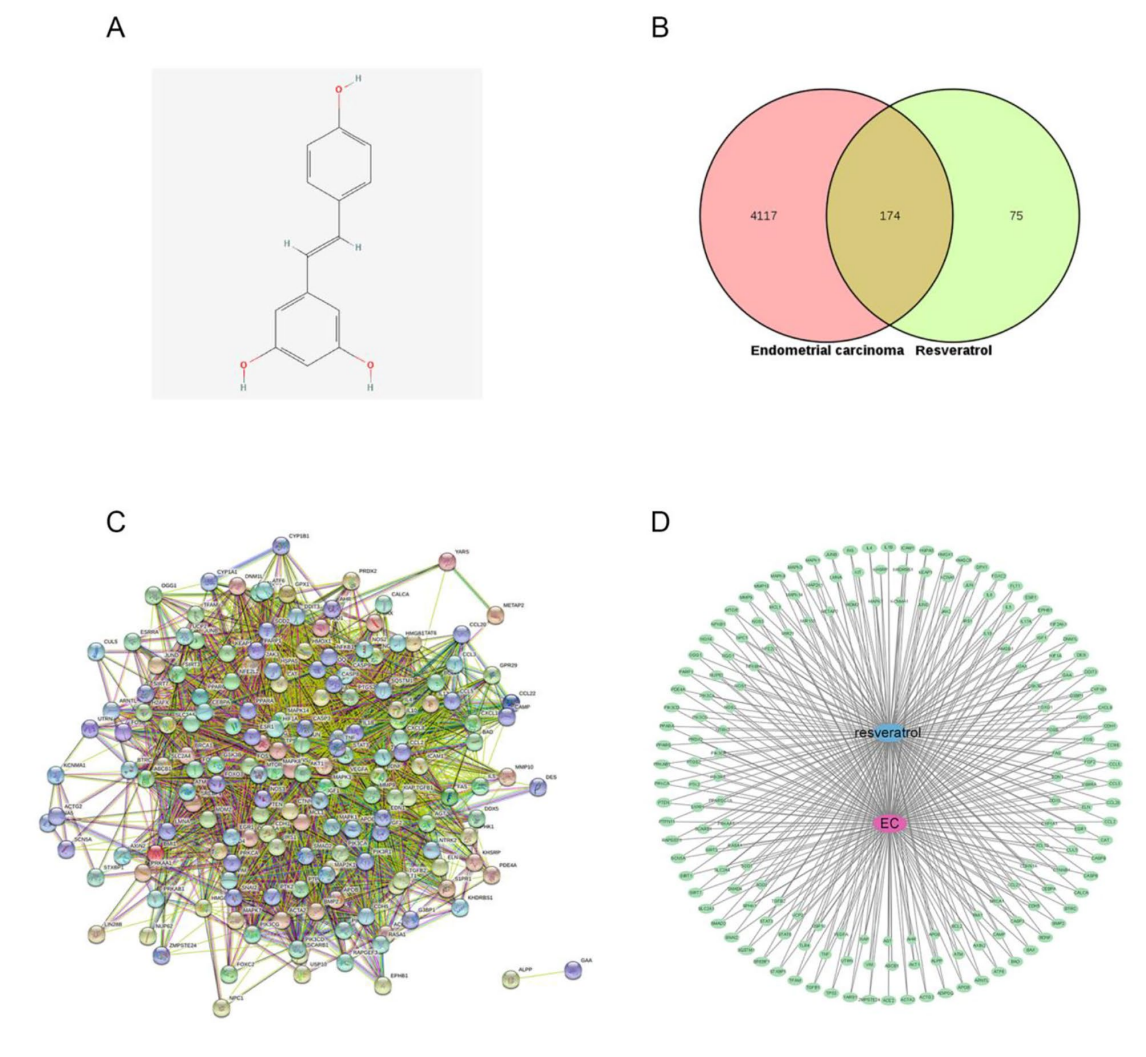
Screening of common targets based on network pharmacology. (**A**) Chemical structure of Res (PubChem CID: 445,154). (**B**) Type I endometrial cancer targets were collected from the Genecards database, and Res targets were collected from the HERB and ETCM databases. After the intersection of the Venn diagram, a total of 174 targets were obtained. (**C**) Protein‒protein interaction analysis (PPI) was performed for the 174 common targets. (**D**) Res‒target‒endometrial cancer network

The chemical structure of Res is shown in Fig. 2A. After data retrieval from the HERB and ETCM databases, 249 targets of Res were obtained after eliminating duplicate genes. Through the GeneCards database, 4291 EC targets were obtained. The intersection of the two was used to obtain 174 potential targets of Res in the treatment of EC, as shown in Fig. 2B. Thus, 174 intersection targets of Res in the treatment of EC were introduced into STRING to obtain the protein‒protein interaction relationship. The interaction network had 173 nodes and 4067 edges, the average node degree value was 47, and the enrichment *P* value was less than 1.0 × 10^− 16^. The data were imported into Cytoscape 3.9.1 to build the Res‒target‒endometrial cancer network, as shown in Fig. 2C and D.

### GO biological function and KEGG pathway enrichment analysis

**Fig. 3:**
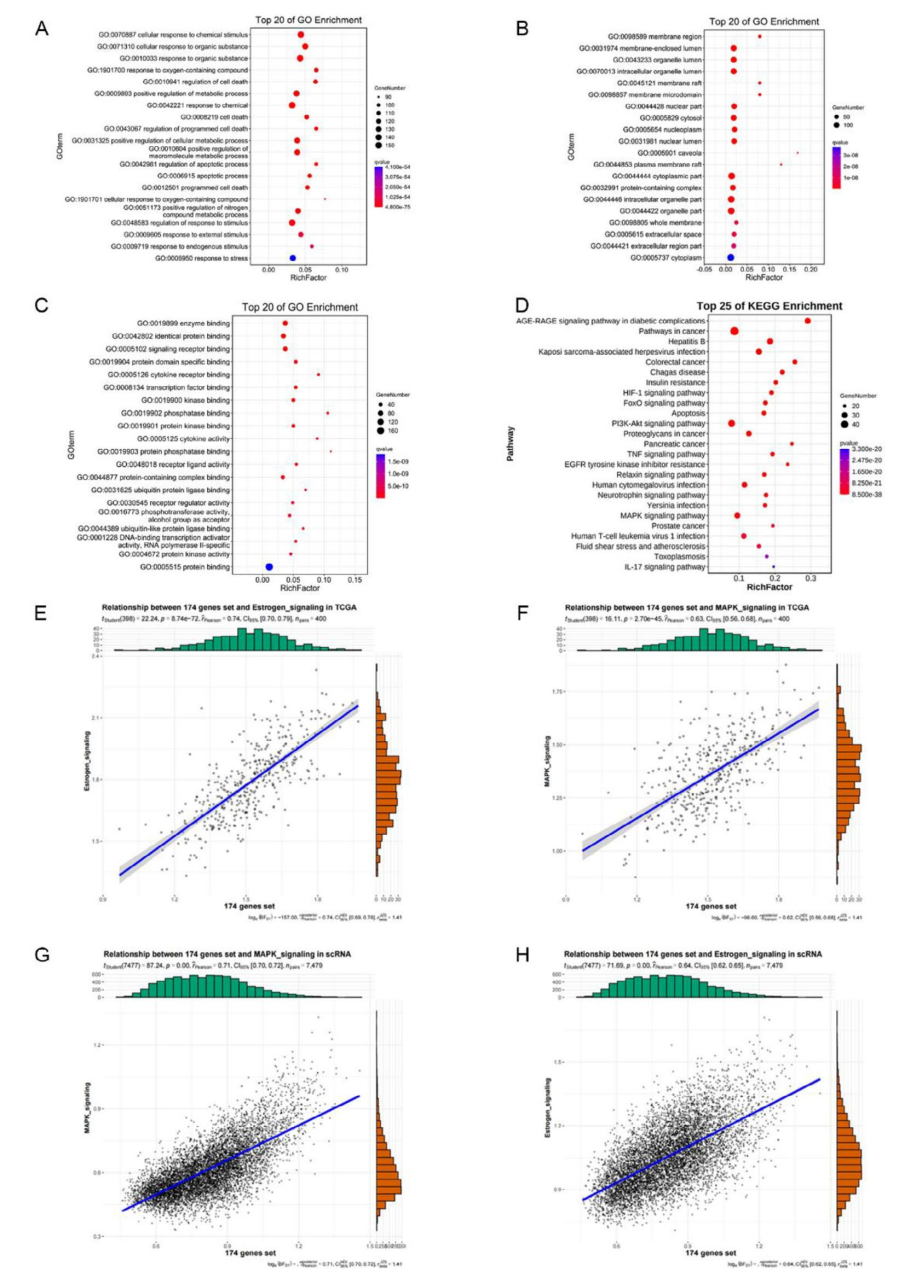
Enrichment analysis of GO and KEGG pathways of key target genes. (**A**) Bioprocess enrichment analysis. (**B**) Cell composition enrichment analysis. (**C**) Molecular function enrichment analysis. (**D**) KEGG enrichment pathway. The size of the circle represents the number of genes, and the colour of the circle represents the corrected *P* value. (**E**) Plot of the correlation between the 174-gene-set score and MAPK gene-set score in the transcriptome data of endometrial cancer in the TCGA cohort. (**F**) Plot of the correlation between the 174-gene-set score and the oestrogen gene-set score in the transcriptome data for endometrial cancer in the TCGA cohort. (**G**) Plot of the correlation between the 174-gene-set score and MAPK gene-set score the in transcriptome data of endometrial cancer in the TCGA cohort. (**H**) Plot of the correlation between the 174-gene-set score and the oestrogen gene-set score in the transcriptome data for endometrial cancer in the TCGA cohort

To illuminate the biological functions and signalling pathways during the process of anti-EC activity of Res, we employed the OmicShare platform to perform GO and KEGG enrichment analysis on the 174 targets. According to the corrected *P* value < 0.05, 6777 biological processes, 544 cell components and 827 molecular functions were obtained. The top 20 enriched terms of BP, CC, and MF were selected and visualized (Fig. 3A-C). Biological processes include mainly cell response to chemical stimulation, response to organic matter, and positive regulation of metabolic processes. The cell components included the membrane region, membrane-enclosed lumen, and organelle lumen. Enzyme binding, identical protein binding and signalling receptor binding were the primary molecular functions.

Furthermore, KEGG pathway enrichment analysis showed enrichment of a total of 213 signalling pathways, mainly involving the cancer signalling pathway, PI3K-Akt signalling pathway, and MAPK signalling pathway. According to their *P* values, the first 25 enrichment data points were plotted as bubble graphs, as shown in Fig. 3. Based on the cellular response to chemical stimuli, the signal transduction function is important, and the MAPK signalling pathway was selected to further explore the mechanism of its action on oestrogen and its metabolites. We then validated the relationship between this 174-gene set and the MAPK signalling pathway, and the results showed a strong positive correlation (*P* < 0.01) between the 174-gene-set score and the MAPK gene-set score in the transcriptome data of endometrial cancer patients in the TCGA cohort (Fig. 3E). Moreover, the results showed a strong positive correlation between the 174-gene-set score and the oestrogen gene-set score (*P* < 0.01) (Fig. 3F). In addition, we also explored the relationship between the 174-gene set and the MAPK signalling pathway at the single-cell level. Similar to the results of bulk RNA sequencing, the 174-gene-set score showed a strong positive correlation with the MAPK signalling score and the oestrogen gene-set score (*P* < 0.01) (Fig. 3G, H).

### PPI network of target genes

**Fig. 4:**
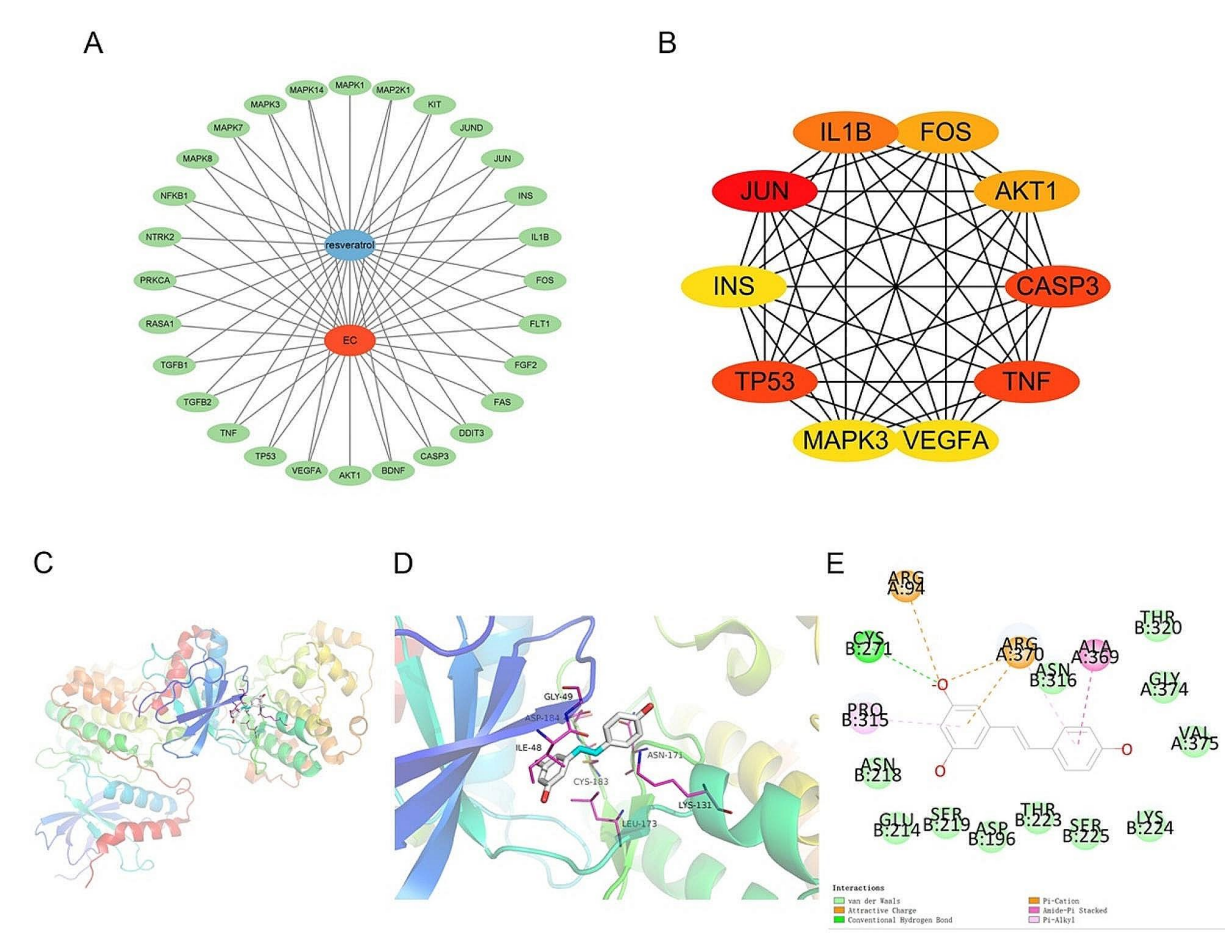
Molecular models of Res binding to its predicted protein targets. (**A**) The target genes with high degree, betweenness and closeness. (**B**) Top ten core therapeutic targets of Res anti-EC. (**C**) Macrograph of three-dimensional docking of Res and MAPK3. (**D**) Micrograph of three-dimensional docking of Res and MAPK3. (**E**) Two-dimensional docking of Res and MAPK3

The targets enriched in the MAPK signalling pathway were input into the STRING database, and the data were imported into Cytoscape 3.9.1. The top 10 core targets of Res for the treatment of endometrial cancer, namely, JUN, TP53, CASP3, TNF, IL1B, AKT1, FOS, MAPK3, INS and VEGFA, were selected according to the degree value, as shown in Fig. 4A and B.

### Docking results analysis

The docking analysis of Res and core target molecules showed that the binding energy of the docking body formed by the core protein TNF and Res was greater than − 5.0 kcal·mol^− 1^. The binding energies of JUN, TP53, CASP3, IL1B, AKT1, FOS, MAPK3, INS and VEGFA with Res were all lower than − 5.0 kcal·mol^− 1^, and the root mean square deviation was less than 2.00, indicating that stable docking could be formed. In addition, the LibDockScore shows that Res can perform semiflexible docking with its receptor ligand of the respective core proteins. Combined with RMSD, chemical energy, and docking fraction, Res formed the most stable docking body with the core protein MAPK3, as shown in Table 1; Fig. 4C-E.

**Table 1:** Docking results of Res with core target molecules

Structural domain	Compound		Vina (kcal·mol-1)	RMSD	DS (LibDockScore)	Hydrogen bond interaction	Hydrophobic interaction
JUN(2GMX)	Res		-7.3	0.292	108.83	GLY:201, TYR:202, GLU:195, SER:179	MET:182, PRO:184, VAL:196
TP53(6UPT)	Res		-7.2	0.209	91.6522	ASP:1536, LEU:1540, ASP:1541, PRO:1537	PRO:1539, PRO:1537
CASP3(2CNO)	Res		-6.5	0.691	91.1883	SER:205, ARG:64, GLN:161, SER:120	ARG:207, CYS:163
TNF(1FT4)	Res		-4.8	0.111	99.0751	PHE:143, GLY:142	CYS:137, CYS:139, CYS:150
IL1B(6Y8M)	Res		-5.4	0.83	48.9654	GLN:48, LYS:94, MET:95	VAL:100
AKT1(3OS5)	Res		-7.2	1.415	93.9267	ASN:263, ASP:256, THR:266, GLY:336	TYR:337, TRP:260
FOS(5FV8)	Res		-5.3	0.232	69.2371	ALA:32, GLN:16, GLN:30	ARG:21, ALA:36, LEU:20
MAPK3(2ZOQ)	Res		-7.4	1.7	89.111	CYS:271	PRO:315, ARG:94, ARG:370, ALA:369
INS(5W2H)	Res		-6.7	1.08	78.1347	ASP:132	ILE:65, VAL:73, LEU:130, ILE:384, LYS:75, ASP:385, TYR:90
VEGFA(4QAF)	Res		-7.7	0.165	79.0837	-	MET:55, VAL:24, VAL:26, VAL:113, VAL:64, VAL:53, ALA:51, ALA:66

### The inhibitory effect of res on the occurrence and development of EC

**Fig. 5:**
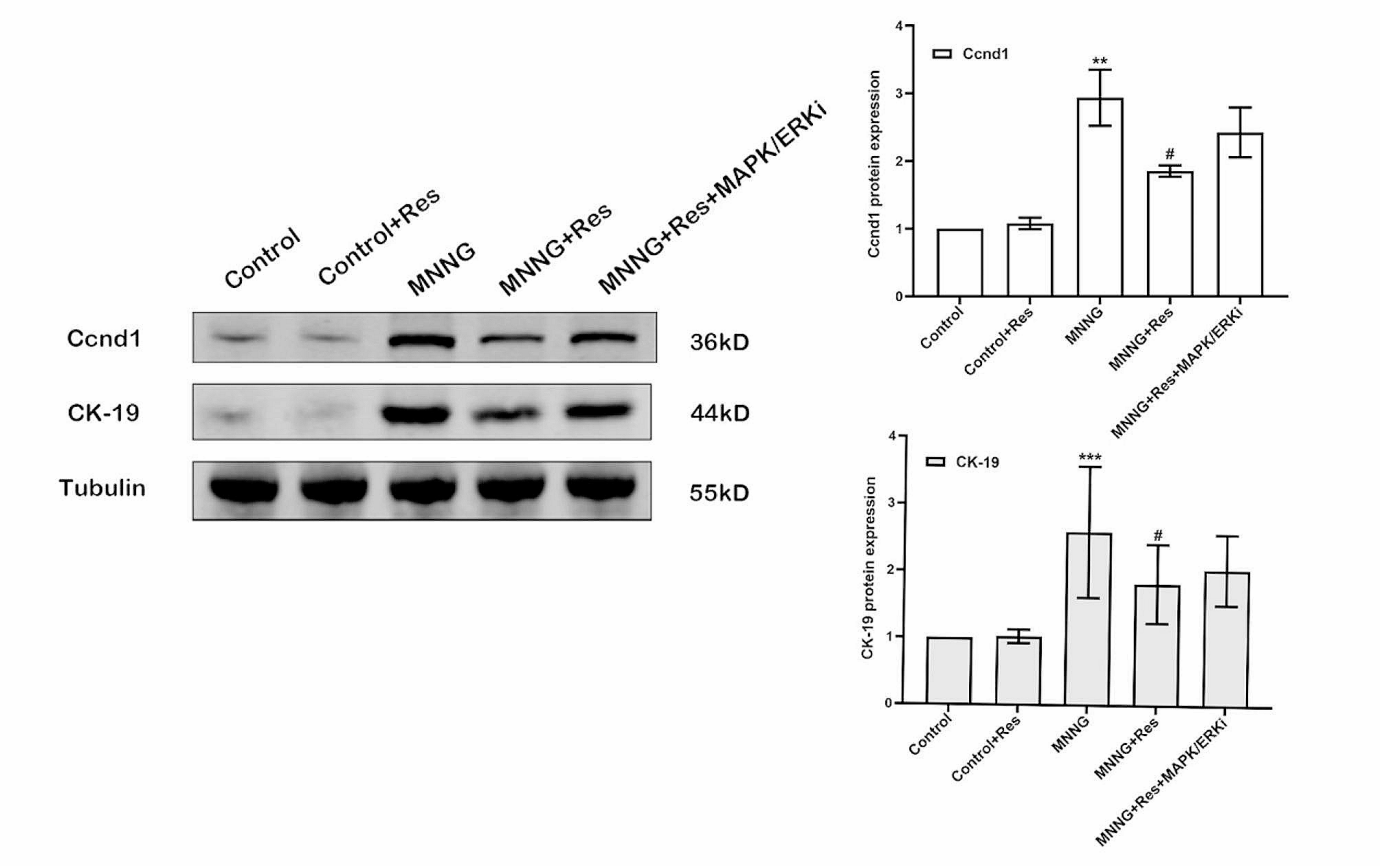
CyclinD1 and CK-19 were significantly elevated in endometrial tissues of model mice compared to Control group. ^**^P < 0. 01, ^***^P < 0. 001 vs. control group; ^#^*P* < 0. 05, vs. MNNG group

As is shown in the Fig. 5, We detected the expression of tumor markers in mouse models by western blot. Compared with the control group, the levels of tumor markers Ccnd1 and CK-19 in the MNNG group were as follows: The protein expression of Ccnd1 and CK-19 were significantly increased (*P*<0.01). After treatment with Res, its expression decreased significantly (*P*<0.01). There were no statistical difference between the MNNG + Res + MAPK/ERK with other group.

### Res increased ERK activation in mouse uterine tissue

**Fig. 6:**
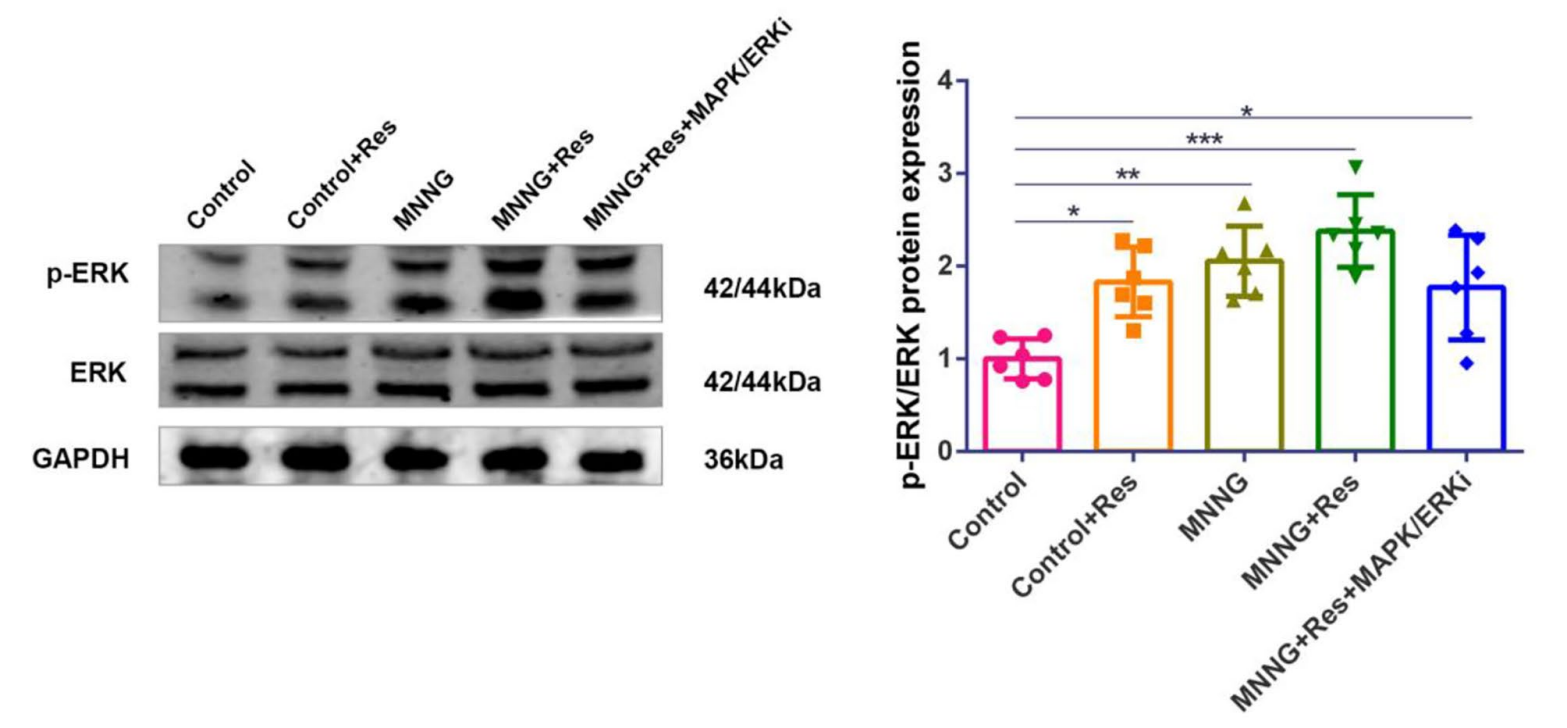
Res increased the level of p-ERK in the endometrial tissue of model mice. There was no difference in the protein level of ERK between groups, and Res increased the protein level of p-ERK. ^*^P < 0.05, compared with control

We conducted experiments to verify whether ERK is the target of Res and check the phosphorylation status of ERK after exposure to Res. Our results showed that ERK phosphorylation was very low in the control group and was increased by Res treatment. In MNNG group ERK phosphorylation was significantly increased. Pretreatment with Res in model mice further increased phospho-ERK levels. Activation of ERK by Res was inhibited when pretreatment with ERKi. However, the difference in p-ERK expression level did not reach statistical significance between MNNG + Res group and the MNNG + Res + MAPK/ERKi group, as shown in Fig. 6. These findings demonstrated that Res administration could activate ERK, and ERKi can partially inhibit the activate effect of Res.

### Res activates the MAPK/ERK pathway to regulate oestrogen homeostasis

**Fig. 7:**
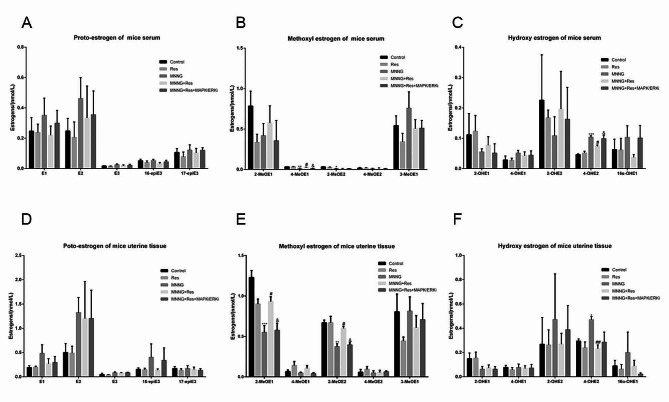
Concentration of oestrogen active substances in the serum and uterine tissue of mice. ^*^P < 0.05, compared with control; ^#^*P* < 0.05, compared with MNNG; ^&^*P* < 0.05, compared with MNNG + Res

To test the hypothesis that Res regulates oestrogen homeostasis by activating MAPK signalling, we used LC-MS/MS analysis to examine the levels of 15 oestrogen and oestrogen metabolites in the serum and uterine tissue samples of mice in the groups. As can be observed compared with the control group, the levels of genotoxicity oestrogens, especially 4-OHE2, in the MNNG group showed a significantly increasing trend in mouse serum and uterine tissues. The levels of protective oestrogen 2-MeOE2 and 4-MeOE1 decreased significantly in the serum of the MNNG group. The levels of protective oestrogen 2-MeOE2 and 2-MeOE1 decreased significantly in the endometrial tissue of the MNNG group. After Res treatment, the homeostasis imbalance was restored, suggesting that Res may have an inhibitory effect on endometrial cancer via regulation of oestrogen homeostasis. In the MNNG + Res + MAPK/ERKi group, the effects of Res on reversing the imbalance of oestrogen homeostasis were significantly weakened (*p* < 0.01; Fig. 7). These findings demonstrate that Res-induced oestrogen homeostasis was achieved through the activation of MAPK signalling pathways.

## Discussion

Elevated levels of oestrogen and its toxic metabolites are crucial factors in the occurrence and development of EC [8, 25]. A growing body of recent literature has highlighted that 2- and 4-hydroxylated catechol oestrogen metabolites can be oxidized into mutagenic quinones that form DNA adducts and lead to DNA damage [26]. In light of the chemoprotective properties attributed to Res by recent epidemiological and animal studies [23], we examined the preventive role of Res and the possible mechanisms by which the anticancer effects of Res against EC occur. Chemically induced EC animal models have been used as translational models to investigate the effects of chemopreventive agents [27]. In the current study, we used MNNG, a nitrosamide, as the chemical carcinogen, and it is a typical DNA damaging agent that inserts methyl groups into several nucleophilic sites of DNA bases, finally causing DNA double-strand breaks [28]. MNNG is a chemical proven to act as a gastric carcinogen in several animal species and hence is widely used to establish animal models of gastric cancer [29]. Later, it was gradually used in gynaecological oncology animal models. The MNNG rat model is an established animal model of EC [30–33]. It shares many features of human EC, such as genomic instability, hormonal imbalance, increased oxidant stress, and DNA damage [31, 33]. In the uteri of MNNG-initiated rats, we observed the expression of the tumour markers Ccnd 1 and CK-19 was significantly increased in the MNNG group (Fig. 5). However, it should be noted that the impact of MNNG on mutagenic changes in various cancer models involves the participation of multiple signalling pathways, which collectively promote the occurrence of malignant tumours and cell proliferation. Therefore, further evidence of the correlation between the MNNG mouse EC model and human EC still requires a substantial amount of research data.

Based on the oestrogen-like effects of Res due to its structural similarity with 17β-oestradiol (E2), some researchers and clinicians are concerned that the intake of Res may negatively affect hormone-dependent malignancies. In fact, in recent studies conducted with both endometrial and mammary cancer cells, scholars have demonstrated that that the antiproliferative and cancer-protective effects of Res occur through an ER-independent pathway [18–20]. Recently, Res has received extensive attention due to its beneficial metabolic regulatory effects. There are many clinical cases that support the potential of Res in improving metabolic function [34, 35]. Two RCTs have investigated the effect of Res on sex hormone levels in patients with polycystic ovary syndrome, and lower serum testosterone, dehydroepiandrosterone sulfate, and luteinizing hormone levels as well as higher follicle-stimulating hormone levels were detected in women treated with Res at 800 or 1500 mg·day^− 1^ [36, 37]. Two other meta-analyses revealed the favourable influence of Res on glycaemic control and insulin sensitivity in diabetic patients [38, 39]. Res seems to target SIRT1, which partly mediates its antidiabetic actions [40]. Another study found that oral treatment with Res at 1 mg per day for 12 weeks increased the concentration of sex steroid hormone-binding globulin (SHBG), resulting in an increased urinary 2-OHE1/16α-OHE1 ratio in postmenopausal obese women. The study suggests that Res has a positive effect on oestrogen metabolism in postmenopausal women and may help prevent the occurrence of breast cancer [41]. In our previous study, we examined the effect of Res on oestrogen metabolism in EC. Our data confirmed that the oestrogen homeostasis profile was disrupted in EC patients and that Res can reverse the imbalance of oestrogen homeostasis. To further elucidate the mechanism through which Res elicits a potentially ameliorative effect on oestrogen homeostasis, we employed network pharmacology approach to screen potential targets of Res in regulating oestrogen metabolism in EC.

Network pharmacology was introduced in the postgenome era to cope with the increasing number of potential targets discovered by the rapid development of pharmacology technology [42]. Traditional Chinese medicine has benefited from this new course of research [43, 44]. A growing number of studies have suggested that Res is a multitarget treatment for EC [9, 19, 45, 46]. In the present study, KEGG pathway enrichment analysis showed that the MAPK signalling pathway is closely related to the targets of Res for endometrial cancer (Fig. 3D). We then validated the relationship between the common target genes and the MAPK signalling pathway and estrogen signalling pathway, and the results showed a strong positive correlation (Fig. 3E-H). Furthermore, we checked the estrogen signalling pathway in the KEGG database, and the graph shows that multiple molecules of the MAPK signalling pathway regulate estrogen signalling. Combined with RMSD, chemical energy and docking score, the docking body formed by the Res and the core protein MAPK3 was the most stable. So we choose MAPK3 for further analysis. MAPK3 also known as ERK, which belongs to the family of mitogen-activated protein kinases (MAPKs), can be phosphorylated and translocated into the nucleus that activating downstream transcription factors to participate in a broad variety of cell functions, including mitosis, metabolism, survival, apoptosis, differentiation and altered gene expression [47–49]. Previous studies have demonstrated that the activation of MAPK pathway is associated with the progression of hormonally driven malignancies, such as EC and breast cancer [50–53]. In addition, the MAPK/ERK pathway also influences chemotherapeutic drug resistance to doxorubicin and paclitaxel in breast cancer cells [54]. However, studies on MAPK/ERK pathway and estrogen metabolites in EC are rarely reported. Then we conducted experiments to verify whether ERK is the target of Res and check the phosphorylation status of ERK after exposure to Res. Our results showed that pretreatment with Res increased phospho-ERK levels, whereas pretreatment with ERKi, inhibited the activation of ERK by Res. However, the difference in p-ERK expression level did not reach statistical significance between Res and the MNNG + Res + MAPK/ERKi group (Fig. 6). The result was in line with our expectations, but not exactly what we had expected. The activation of ERK may be multifactorial. One of the reasons for the above results may be the reciprocal cross-talk between MAPK pathways in response to stimuli. In general, the cross-talk between MAPK pathways is mediated by a complex network of protein phosphatases [55]. These phosphatases are negative feed-back regulators that are usually induced by the upstream activators at different levels and cross-react with substrates in more than one signalling channels. Responses to stimuli may depend at least in part on the dynamic balance between MAPK pathways [56]. Therefore, it may be reasonable to describe MAPK signals more accurately, better understand the pathway feedback loop, and ultimately use this information in the rational design of research ideas to target multiple factors in the pathway or simultaneously target other pathways.

To confirm that Res regulates estrogen metabolism via MAPK/ERK signalling pathways, we used LC-MS/MS to examine the levels of 15 oestrogen and oestrogen metabolites in the serum and endometrium tissues of mice in the groups. Our results show that the levels of proto-estrogen, including E1, E2, E3, 16-epiE3 and 17-epiE3 do not change significantly in different groups both in serum and endometrium tissues, whereas the carcinogenic hydroxy-oestrogen 4-OHE2 increased significantly and methoxyl oestrogens 2-MeOE1, 2-MeOE2,4-MeOE1 decreased in model mice both in the serum and endometrium tissues (Fig. 7). The results revealed that the oestrogen homeostasis profile was disrupted in EC model mice. These results are not entirely consistent with the epidemiological characteristics of patients with EC. In EC patients, carcinogenic hydroxy-oestrogen (4-OHE1, 2-OHE1, 2-OHE2) significantly accumulated and methoxyl oestrogens do not change significantly in the serum [8]. Due to scarce available data, it is unclear if the discrepancy is associated with species variation, or owing to the large inter- and intra-individual variations in sex hormone concentration during the menstrual cycle [23, 57]. However, the levels of 4-OHE2 reduced to normal level after Res treatment both in serum and in endometrium tissues. These results indicated that the target tissue of EC showed a notable accumulation of hydroxy-oestrogen, supporting an accumulation of toxic estrogen species 4-OHE2 in the endometrium tissues, and Res can facilitate estrogen homeostatic reprogramming via the hydroxy-oestrogen pathway. When pretreatment with ERKi, Res failed to reprogram oestrogen homeostasis in serum, demonstrating that the protective effect of Res may be related to the activation of the ERK/MAPK signalling pathway (Fig. 7C). Likewise, in endometrium tissues, ERKi also inhibited the effects of Res on restoring estrogen homeostasis although the difference was not statistically significant (Fig. 7F). In addition, our study also showed that endometrium tissue catechol estrogens level was three to four times higher than in the serum. There are evidences that different lesion sites show distinct local estrogen concentration [58], which, in turn, might be due to their unique local estrogen metabolism. Oestrogen homeostasis may also be mediated by other factors that are independent of MAPK signalling. Additionally, the mechanism(s) of Res-mediated prevention of EC could be more complicated than currently understood. The mechanism of antitumor activities of Res on EC needs more evidence and further study.

Although Res has been previously reported to have anticancer effects in EC, this study was the first to show that Res activates the MAPK/ERK pathway to regulate oestrogen metabolism in EC. MAPKs are protein kinases that mediate extracellular signal transduction to intracellular signalling and play an important role in various physiological processes [52]. Members of the MAPK family include ERK, JNK and p38 MAPK [59]. Moreover, the MAPK/ERK pathway is activated by several external stress factors, such as hyperglycaemia, haemodynamic abnormalities, oxidative stress and proinflammatory cytokines [60]. These findings supported our hypothesis that, under the accumulation of toxic metabolites of oestrogen hydroxylation, Res activating the MAPK/ERK pathway, cells perceive the imbalance of oestrogen metabolism homeostasis, altering other messenger molecules and modifying cell metabolism [61]. Metabolizing enzymes in the body are an important means to regulate the dynamic balance of estrogen metabolism [61]. The exact relationships between Res, MAPK, and oestrogen metabolism enzymes in EC will be evaluated in our future research.

In conclusion, the current study indicates that Res can activate ERK phosphorylation in EC model mice, thus reducing the accumulation of hydroxylated oestrogen and restoring the homeostasis of oestrogen. The MAPK/ERK pathway is a promising potential target for the treatment of imbalances in oestrogen metabolism homeostasis. These findings provide new evidence to better understand the antitumor activity of Res that could facilitate further investigation of its potential use in the clinical setting.

### Electronic supplementary material

Below is the link to the electronic supplementary material.

Supplementary Material 1

## Data Availability

The authors appreciate the availability of any data used in this study. All data and material will be made available on request. The data used in the present study are available from the corresponding author upon reasonable request.
