# Supplementary material for: Resveratrol activates MAPK/ERK pathway to regulate oestrogen metabolism in type I endometrial cancer
Source: BMC Complement Med Ther. 2024 Jun 11;24:227. doi: 10.1186/s12906-024-04509-y (PMC11167854; doi:10.1186/s12906-024-04509-y)
Supplement: Supplementary file 1 — Supplementary Material 1 [file 12906_2024_4509_MOESM1_ESM.docx]

Dear editor, The original datas of western bolt in the picture below.

p-ERK (42/44KDa)

ERK (42/44KDa)
